# Factors affecting oral and dental services` utilization among Elderly: a scoping review

**DOI:** 10.1186/s12903-023-03285-4

**Published:** 2023-08-27

**Authors:** Mohadeseh Ghanbari-Jahromi, Peivand Bastani, Faride sadat Jalali, Sajad Delavari

**Affiliations:** 1 Student Research Committee, Shiraz University of Medical Sciences, Shiraz, Iran; 2 College of Health and Human Sciences, Faculty of Health, Charles Darwin University, Darwin, Australia; 3 Health Human Resources Research Center, School of Health Management and Information Sciences, Shiraz University of Medical Sciences, Shiraz, Iran

**Keywords:** Utilization of health services, Oral and dental, Elderly, Scoping review

## Abstract

**Background:**

Regular use of oral and dental services by the elderly is one of the important factors in reducing oral and dental diseases. This study aimed to identify the factors affecting oral and dental services` utilization among elderly.

**Methods:**

The published articles on the factors affecting oral and dental services` utilization among elderly were found through a scoping search and using related keywords in PubMed, Scopus, Embase, and Web of Science databases within January 2000 - December 2022 according to the PRISMA guidelines. The data were analyzed using the thematic analysis method.

**Results:**

Among the 2381 articles retrieved from the databases, forty-two were extracted. The factors affecting oral and dental services` utilization among elderly were classified into five main components as follows: access, demographic factors, social factors, health level, and mental factors. The results showed that income, education level, living area, number of teeth, and importance of care were the most frequent in the main components of access, demographic factors, social factors, health level, and mental factors, respectively.

**Conclusion:**

Equitable utilization of oral and dental services is the right of all members of the society, especially the elderly. Therefore, it is necessary to provide the elderly with suitable conditions to utilize such services, which are mostly luxury items. Furthermore, increasing the elderly’s awareness and encouraging them to use oral and dental services regularly can help reduce the burden of oral and dental diseases.

**Supplementary Information:**

The online version contains supplementary material available at 10.1186/s12903-023-03285-4.

## Introduction

The demographic changes caused by old age and decreased birth rates made a transformation in the age structure of the world’s population [1]. It is predicted that by 2030, one in six people in the world will be 60 years old or above;and by 2050, the population of people over 60 years old will reach about 2.1 billion [2].

As the number of elderly people increases, the number of people with oral diseases increases [3]. About 3.5 billion people worldwide suffer from oral diseases [4], the exact number of elderly people suffering from it is not known [5]. Oral health of the elderly has become a global concern. The most common of these are periodontal disease, tooth decay, tooth loss, xerostomia, and oral pre-cancerous and cancerous conditions [6, 7]. Complications caused by oral diseases including pain, infection, and loss of function can lead to reduced quality of life and reduced use of dental services by the elderly [8]. Due to the importance of dental services for the elderly, regular use of these services is very important and necessary. According to Brown et al. (1999), the utilization of dental services refers to the percentage of the population that has access to these services at a certain period of time [9]. Regular visits to the dentist lead to timely diagnosis and treatment of oral diseases, as well as health promotion, oral health education, and maintenance of oral health status[10]. However, older people are less likely to seek dental care than others[11]. According to a study, the use of dental services peaks during adolescence and decreases in old age [12]. In other words, the number of visits to the dentist decreases with age in the elderly age [13]. At the age of 60–70 years, people face problems such as loss of income due to not being active labor force, increased probability of chronic diseases, loss of social support, and inadequate pensions affecting their health and oral health [14]. Also, poor oral health has a negative impact on nutrition and general health [15, 16].

According to the previous literature, there is no single barrier to accessing dental health care for the elderly [12], and various factors affect the utilization of these services by the elderly. For example, the results of the study by Mariño et al. (2014) showed that the elderly in Victoria’s rural areas used dental services less within 12 months in 2014 [17]. According to Choi et al. (2020), with the decrease in household income, the use of dental services and dental implants in South Korea’s elderly is reduced [18]. Smith et al. (2020) also showed that the cost, fear, and childhood experiences were important barriers to the lack of dental care in the New Zealand elderly [19]. The study by Zhang et al. (2019) in the United States showed that elders who are under insurance are more likely to use dental services than those without health insurance [20]. The results of Drachev et al. (2022) also showed that higher education, pain or discomfort in the tooth/mouth, and fewer missing teeth, are associated with more dental use in the Lithuanian elderly [21].

Due to the inequalities in the oral and dental health of the elderly in different societies, it is very important to identify the factors influencing the use of oral and dental services. Considering this issue, studies have been done scattered all over the world. But the researchers could not find a study that extensively and comprehensively examines these factors during their investigations. So, the question was raised, what factors affect the use of these services by the elderly in the world? Conducting a comprehensive study on the factors affecting the elderly’s use of oral and dental services can help governments, health policymakers, and insurance companies in designing comprehensive oral and dental health programs and reducing their treatment costs. Therefore, this study was conducted to determine the factors affecting the utilization of dental services in the elderly through a scoping review.

## Materials and methods

This study is a scoping review, the framework of which was adopted from the scoping review method of the Joanna Briggs Institute [22]. Thus, determining the factors affecting oral and dental services` utilization among the elderly was carried in the following five stages:

In the first stage, a specific question was asked based on the PCC elements (population, content, and context) as follows: the elderly over 50 years of age (population), the factors affecting utilization of oral and dental services (concept), and the economic, social, cultural, and health conditions in different countries of the world (context). The research question was “What are the factors affecting the utilization of oral and dental services by the elderly?”

The second stage was conducting a review of the literature, including all studies on the “utilization of oral and dental services” in different countries. To this end, all relevant studies conducted since 2000 were retrieved through the search strategy (Table 1). Therefore, the appropriate and relevant keywords to the research objective were selected and searched in PubMed, Scopus, ISI Web of Science, and Embase databases. Finally, all the articles with at least English abstracts indexed in the databases (n = 2381) were identified and retrieved. After eliminating the duplicates, the remaining 1338 articles were reviewed by titles. Furthermore, 413 articles were reviewed in terms of abstracts and 93 were retrieved by full texts to check if they met the inclusion criteria. In the end, 42 articles were included in this study.

**Table 1:** The search strategy of the research

Database	Search string	limits	date	Number of retrieved papers
SCOPUS	( TITLE-ABS-KEY ( aged ) OR TITLE-ABS-KEY ( elder* ) OR TITLE-ABS ( older* ) ) AND ( TITLE-ABS-KEY ( "health care utili*" ) OR TITLE-ABS-KEY ( "healthcare utili*" ) OR TITLE-ABS-KEY ( "health service utili*" ) OR TITLE-ABS-KEY ( "health service use" ) OR TITLE-ABS-KEY ( "health care use" ) OR TITLE-ABS-KEY ( “healthcare use” ) OR TITLE-ABS-KEY ( “service utilization” ) OR TITLE-ABS-KEY ( “service use” ) OR TITLE-ABS-KEY ( "health service utilization" ) OR TITLE-ABS-KEY ( “healthcare utilization” ) OR TITLE-ABS-KEY ( "health care utilization" ) ) AND ( TITLE-ABS-KEY ( dental ) OR TITLE-ABS-KEY ( oral ) TITLE-ABS-KEY ( teeth ) OR TITLE-ABS-KEY ( tooth ) OR TITLE-ABS-KEY ( dentist* ) )	Language to English	Up to December 2, 2022	427
PUBMED	((Aged[Title/Abstract] OR elder*[Title/Abstract] OR older*[Title/Abstract]) AND (“health service use“[Title/Abstract] OR “health care use“[Title/Abstract] OR “healthcare use“[Title/Abstract] OR “service utilization“[Title/Abstract] OR “service use“[Title/Abstract] OR “health service utilization“[Title/Abstract] OR “health care utili*“[Title/Abstract] OR “healthcare utili*“[Title/Abstract] OR “health service utili*“[Title/Abstract] OR “health care utilization“[Title/Abstract] OR “healthcare utilization“[Title/Abstract])) AND (dental[Title/Abstract] OR teeth[Title/Abstract] OR tooth[Title/Abstract] OR oral[Title/Abstract] OR dentist*[Title/Abstract])	Language to English	Up to December 2, 2022	485
WOS	(TS=(Aged OR elder* OR older*) AND TS=(“health care utili*” OR “healthcare utili*” OR “health service utili*” OR “health service use” OR “health care use” OR “healthcare use” OR “service utilization” OR “service use” OR “health service utilization” OR “health care utilization” OR “healthcare utilization” ) AND TS=(Dental OR oral OR tooth OR teeth OR dentist*))	Language to English	Up to December 2, 2022	836
EMBASE	(‘aged’:ab,ti OR ‘elder*’:ab,ti OR ‘older*’:ab,ti) AND (‘health care utili*’:ab,ti OR ‘healthcare utili*’:ab,ti OR ‘healthcare use’:ab,ti OR ‘health service utili*’:ab,ti OR ‘health service use’:ab,ti OR ‘health care use’:ab,ti OR ‘service utilization’:ab,ti OR ‘service use’:ab,ti OR ‘health service utilization’:ab,ti OR ‘health care utilization’:ab,ti OR ‘healthcare utilization’:ab,ti) AND (‘Dental’:ab,ti OR ‘oral’:ab,ti OR ‘teeth’:ab,ti OR ‘tooth’:ab,ti OR ‘dentist*’:ab,ti)	Language to English	Up to December 2, 2022	633
Total				2381

**Fig. 1:**
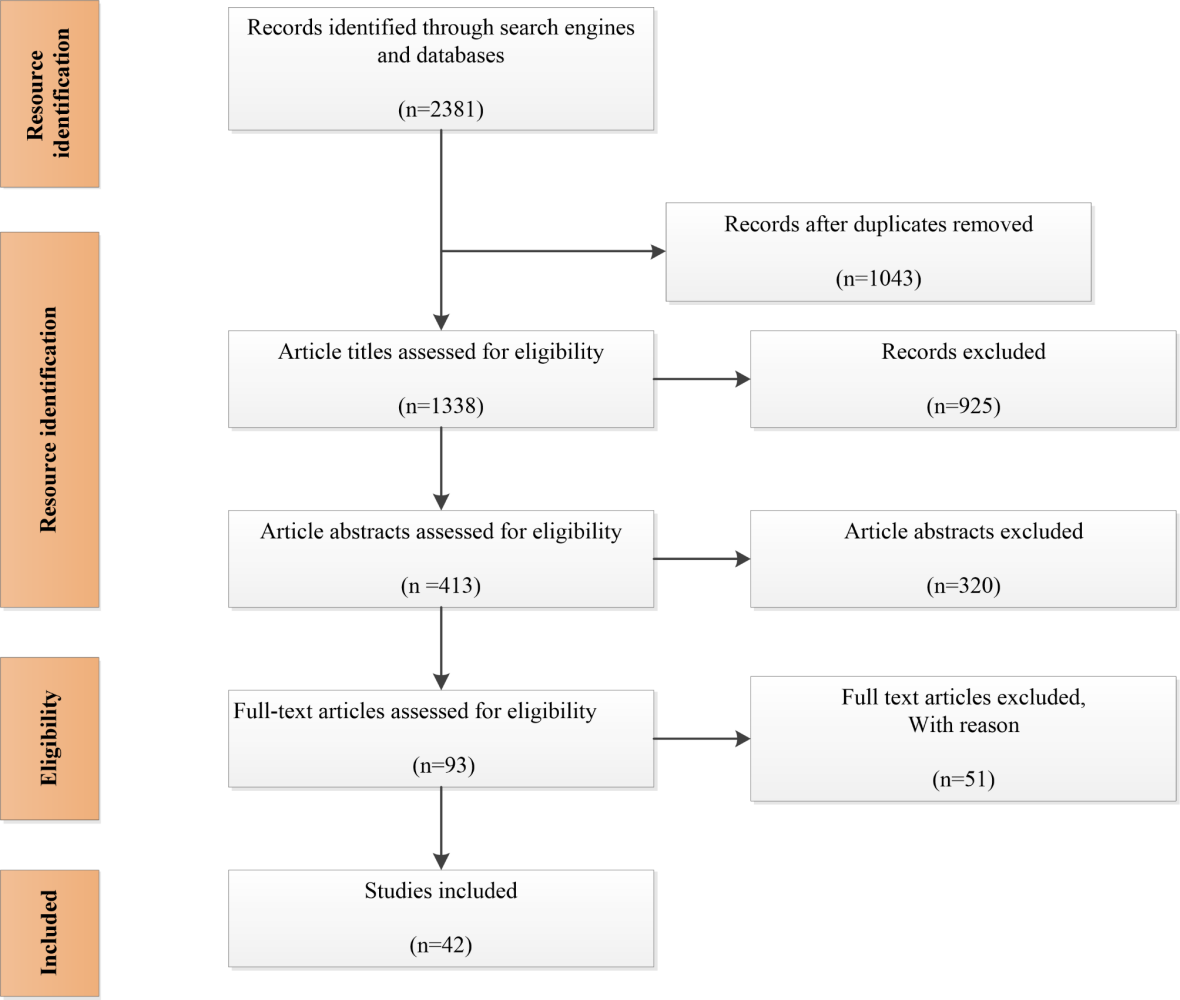
Preferred Reporting Items for Systematic Reviews and Meta-Analyses (PRISMA) flow diagram for the scoping review process

The selection of studies related to the topic was done in the third step (Fig. 1). It should be noted that all the stages of research and selection of articles were done by two researchers independently (MGH and FSJ), and in case of disagreement, a third researcher (SD) was used for consensus. Review studies, letters, commentaries, and articles whose research populations included people under 50 years of age were excluded. Finally, the CASP technique was used to measure the quality of original articles.

In the fourth stage, a data-charting form (Appendix Table A1) was used to extract the data from each study. Thus, names of the first authors, titles, years, place, types, participants, and the results of the studies were recorded in the data extraction form on the Microsoft Excel software. Finally, the data obtained from the previous stage were classified using the thematic analysis method. Hence, the data collected in the previous stage were open coding based on the research question. After re-checking the codes as well as finalizing and reducing them, the process of categorizing and aggregating the codes continued until obtaining sub-components and main components that determined the utilization of oral and dental health services among the elderly. The results were then entered in a table (Table 2).

**Table 2:** Factors affecting oral and dental services utilization among elderly

Main component	Sub component	Reference	Reference evidence
**Access**	Length of waiting list	[17, 39]	Utilization of dental services had a negative relationship with the elderly’s length of waiting list.
	Access level	[19, 35, 76]	Access had a positive relationship with utilization of oral and dental services by the elderly.
	Income	[17, 18, 20, 23, 24, 28, 39, 50, 51, 57, 69, 75, 77, 79, 82, 94–99]	Income had a positive relationship with utilization of oral and dental services.
	Membership in Insurance Funds	[20, 28, 29, 52, 57, 62, 69, 77, 82, 98–101]	Utilization of oral and dental services was higher among the elderly membered in insurance funds.
	Financial Costs	[17, 19, 39, 47, 76]	Utilization of oral and dental services by the elderly decreased due to high costs.
**Demographic Factor**	Age	[13, 20, 24, 39, 51–53, 57, 58, 75, 79, 96, 97, 102]	Age had a negative relationship with utilization of oral and dental services.
	Gender	[19, 20, 52, 57, 58, 69, 77, 79, 96]	Most of the studies showed that utilization of oral and dental services was higher among elderly women than men. However, according to Smith et al.‘s study, elderly men also regularly utilized dental services [19].
	Education level	[8, 17, 18, 20, 23, 24, 28, 29, 39, 42, 50–52, 57, 69, 75, 77, 79, 82, 95–97, 101]	Utilization of oral and dental services was higher among the elderly with higher education levels
	Marital Status	[17, 20, 57, 58, 97]	Married elderly people used dental services more.
	Race/ethnic	[20, 24, 62, 63, 97]	Non-white elderly people had lower utilization of dental services.
**Social Factors**	living Area	[24, 28, 50, 52, 53, 69, 97, 102, 103]	Utilization of oral and dental services was lower among the elderly living in rural areas.
	Migration	[39, 47, 73, 75, 76, 101]	Migration had a positive relationship with utilization of oral and dental services.
	Family and Social Support	[19, 35, 50, 57, 73, 75, 101]	Most studies showed that family and community support had a positive relationship with utilization of oral and dental services. But no relationship was found in the study by Wu et al. [75].
	Working Groups	[17, 39, 77]	The elderly people who worked in low-level working groups had less utilization of dental services.
**Health level**	Number of Teeth	[8, 17, 23, 24, 39, 42, 50, 58, 75, 79, 82, 104]	Number of teeth had a negative relationship with utilization of oral and dental services.
	Smoking	[20, 28, 51, 57, 75, 99]	In all studies, there was a negative relationship between smoking and utilization of oral and dental services, except for Zhang et al.‘s study [20].
	Physical problems	[17, 20, 50, 51, 57, 79]	Utilization of oral and dental services was lower among the elderly with physical problems.
	Need to treatment	[17, 23, 24, 42, 73, 82, 101]	According to most studies, utilization of dental services was higher among the elderly who needed treatment. But Manski et al. had reported a negative relationship [82].
**Mental factors**	Importance of Care	[8, 17, 35, 82]	Importance of care had a positive relationship with utilization of oral and dental services.
	Traditional beliefs	[12, 19, 35]	Utilization of dental services was lower among the elderly who had traditional beliefs about oral and dental care.
	Fear	[12, 17, 45]	Fear had a negative relationship with utilization of oral and dental services.
	Previous Experience	[12, 19]	The elderly people who had negative experiences of dental services visited dentists less often.
	Awareness of Care	[12, 19, 39, 77]	The elderly people, who were more aware of their dental health, were more likely to utilize dental services.

## Results

**Fig. 2:**
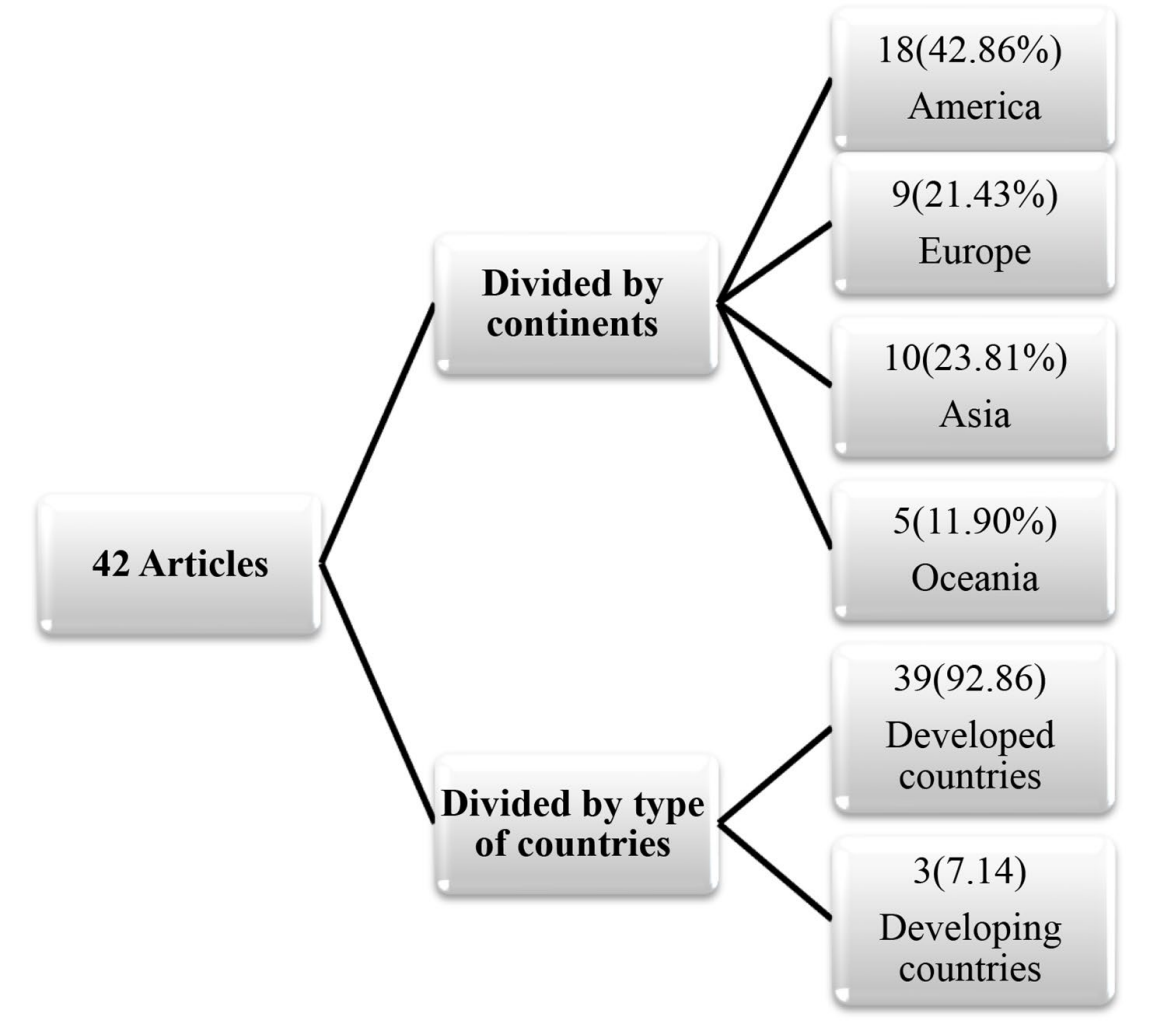
Classification of articles by continent and type of countries

The results of analyzing 42 reviewed studies are summarized in Appendix Table A2. Of those, 18 (42.86%), 10 (23.81%), 9 (21.43%), and 5 (11.90%) studies were related to America, Asia, Europe, and Oceania continents, respectively. In addition, 39 (92.86%) and 3 (7.14%) studies were related to developed and developing countries, respectively (Fig. 2).

Other findings showed that five main components were emphasized as the factors affecting oral and dental services` utilization among elderly, including access, demographic factors, social factors, health level, and mental factors (Table 2).

As Table 2 shows, 31 articles (73.81%) mentioned demographic factors as one of the main components in utilization of oral and dental services by the elderly. The five sub-components included education level 24(42/11%), age 14(24.56%), gender 9(15.79%), race 5(8.77%), and marital status 5(8.77).

The second most referred main components were access, mentioned in 30 articles (71.42%). The five sub-components included income level 21(47.73%), insurance funds membership 13(29.55%), access level 3(6.82%), financial costs 5(11.36%), and Length of waiting list 2(4.55%).

The number of articles on “social factors” was 20 (47.62%). This main component that affected utilization of oral and dental services by the elderly had four sub-components as follows: living area 9(36%), family and social support 7(28%), migration 6(24%), and working groups 3(12%).

Health level was another main component addressed in 19 articles (47.5%). The sub-components of this theme included the number of teeth 12(38.71%), need for treatment 7(22.85%), smoking 6(19.35%), and physical problems 6(19.35%).

Finally, the least referred were related to the main components of Mental factors”, which mentioned in nine articles (21.43%). Importance of care 4 (25%), awareness of care 4 (25%), traditional beliefs 3 (18.75%), fear component 3 (18.75%), and past experiences 2 (12.5%) were its sub-components.

Additional information on the relationship between utilization of oral and dental services among elderly and the sub-components identified in this study is summarized in the Table 2.

## Discussion

Dental services are among the most important health services for the elderly, neglecting which might affect their health [14] and increase their costs [19]. According to the results, the main component most frequently mentioned in the articles was demographic factors, whose most referred sub-component was education level. The most referred sub-components in the main components of access, social factors, health, and mental factors were income level, living area, number of teeth, and importance of care, respectively. In the following, the components influencing utilization of oral and dental services by the elderly are discussed in detail.

The studies mostly addressed the sub-component “income level” in the main component “access”. According to the studies by Soares (2021), Meneses (2016), and Ferreira et al. (2013), as income increased, the proportion of the people who used oral and dental services increased as well [23–25].

In their study, Cheng et al. (2018) also reported in their study that household income has a positive relationship with the utilization of oral health services [26]. However, a study indicated that income alone did not determine utilization of dental services, and other factors such as the individual’s wealth could be effective [27]. The elderly often used less oral and dental services because they had little or no income after retirement [28]. Hence, according to the results of the studies, the higher the people’s affordability, the more favorable conditions would be provided for utilization of oral and dental services.

Membership in insurance funds was another sub-component of this study. According to the studies by Archuleta et al. (2022), Manski et al. (2016), and Somkotra et al. (2013), having dental services insurance and continuous coverage of people increased utilization of these services [29–31], the reason for which could be more services that insurance packages provided to the elderly [28]. In many countries, the performance of the government and support organizations in increasing the insurance coverage of oral and dental services was insufficient [32–34]. Therefore, convincing insurance companies to cover oral and dental services for the elderly could help increase utilization of such services.

One of the barriers to utilizing oral and dental services was the financial cost. This is in line with the results of the studies by Smith et al. (2020) and Mariño (2014) [17, 19]. In other words, dental services imposed an additional economic burden on households and could eliminate their ability to pay and their motivation to use the services [24]. Therefore, allocating budgets or government subsidies to oral and dental services could increase not only oral and dental health levels but also utilization of dental services by the elderly.

Access level was one of the components that could make utilization of oral and dental services difficult. The results of the studies showed that the lack of transportation means or people who could take the elderly to dental clinics was one of the obstacles to access dental care [19]. In other words, as people got older and developed cognitive and physical problems, it would be harder for them to drive and they used public transportation more often [35–38]. It seems that access was an important factor in utilization of oral and dental services, and the services could be increased by taking necessary measures to improve the elderly’s access levels.

Length of waiting list was another factor mentioned in the studies. The studies showed that long waiting lists could reduce people’s desire for regular visits and utilize dental services [17, 39]. However, a study showed that different expectations of patients might have a great impact on the perception of waiting time [40]. It seems that planning an appointment system could provide more appropriate conditions for the elderly to utilize oral and dental services. Additionally, providing some remote dental services could reduce the waiting time for appointments, triage, and prioritization of visits [41].

Considering demographic factors, education level was the most referred sub- component in the studies. According to the studies by Drachev et al. (2022), Ren et al. (2020), and Saleh et al. (2018), higher levels of education was associated with the increased number of people who utilized dental services, due to their greater awareness of the importance of regular use such services [23, 42–44]. It seems that people with higher education levels had better health habits to maintain their oral and dental health. However, some studies showed that education level had no relationship with utilization of oral and dental health services [45, 46]. The dispersion in the results could be due to the difference in the populations of these studies and the individuals’ behavioral characteristics.

Gender was another important demographic factor. Some studies showed that women had a greater desire to preserve and maintain their teeth than men and had more referrals to medical centers [20, 47–49]. However, many men also cared about the appearance of their teeth and visited dentists regularly. Thus, the stereotype that women are more interested in the appearance of their teeth than men might not be true and depends more on individual factors [19].

Age was another factor addressed in the studies. It was found that, due to the great needs of the elderly for oral and dental care, they were less inclined to use dental services [50–53]. Janto et al. (2022) stated that this was due to the deterioration of the elderly’s general health [54], mobility problems, anxiety, and lack of awareness about dental health during dental visits [55, 56]. Therefore, providing education and active care for the elderly seems necessary.

Marital status was also mentioned in the studies. Zhang et al. (2019) reported that regular utilization of dental services was more frequent among married people, and other studies confirmed it as well [20, 57–59]. Married people often paid more attention to dental care due to their greater commitment and responsibility towards their spouses as well as their social control role of their spouses [57, 60]. However, a study showed that there was no significant relationship between marital status and utilization of dental services [61]. The difference could be due to the different community of that research and the age range of its participants.

Race or ethnicity was another factor investigated in the studies. Wu et al. (2022) and some other studies showed that the non-white elderly used oral and dental services less often [62–65]. Also, Amanat et al. (2020) suggested that tooth decay and tooth loss in the Chinese elderly was less frequent than in the Malay elderly [66]. Financial problems, racial discrimination, differences in oral and dental health literacy, perception of needs, barriers to access, and dissatisfaction with dental care among elderly people of different races could be the reasons for it [63, 67]. However, a study showed that there was no significant relationship between different ethnic groups and periodontal health status [68], which could be due to the difference in the age groups of the subjects studied.

The most important sub-component of the social factors was living area. Harirugsakul (2020) et al. and several other researchers reported that the old people in urban areas were more inclined to use dental services than in rural areas [24, 28, 30, 69]. This could be due to transportation problems and lack of dentists in rural areas [70–72]. Therefore, it seems that government financial support/subsidies are necessary for better access of the elderly and providing suitable conditions for the presence of dentists in rural areas.

Social support was one of the other influential components in the studies reviewed. Smith et al. (2020) and Lai et al. (2007) stated that support from family was one of the facilitators of access to and utilization of dental care by the elderly [19, 73]. According to the study by Niesten (2013) in the East of the Netherlands, elderly people often stopped their oral care due to confusion and lack of social support [35]. In fact, social support for the elderly could sometimes help to develop behaviors related to oral health and lifestyle modification[74]. However, according to Wu et al.‘s (2005) study on Chinese and Russian immigrant elderly, social support, which included visiting friends and family members by the elderly in Russian, did not have a significant effect on dental services utilization, unlike the Chinese elderly [75]. This could be due to the difference in the individual and behavioral characteristics of the studied subjects.

Migration and its consequent problems had a great impact on utilization of dental services by the elderly. According to some studies, elderly migrants used fewer oral and dental services [47, 67, 76]. The costs, language problems, and lack of trust in dental services were barriers to dental care among elderly migrants [47]. Length of stay was also effective in migrants’ utilization of services. Referring to Wu’s (2005) study, the probability of visiting a dentist increased by 7% each additional year of residence in the United States [75]. It seems that social factors like individual characteristics and behaviors of the elderly were effective in this regard.

Working groups of the elderly was considered as another factor affecting utilization of oral and dental services by them. Studies showed that people working in low-level working groups or those who had reached the retirement age in lower working groups utilized dental services less frequently [17, 39, 77]. Indeed, people in insecure working groups and those who were not employed were more likely to have unmet dental care needs than others due to the economic burden.

Number of teeth was the most important sub-component of health level in the studies investigated. Toothless elderly people used less oral and dental services (22). In several studies, the correlation between the number of teeth and utilization of regular dental services was high [23, 42, 77]. However, the study by Yuan et al. in 2020 showed that Chinese elderly people who had fewer or no teeth had more referrals to the centers providing dental services in order to receive dental prostheses or denture services [78]. The difference in the results could be due to individual characteristics, knowledge, and attitudes of the people participated in the studies.

Smoking had been addressed in the studies as an effective factor in the elderly’s utilization of oral and dental services. In their studies, Moeller et al. (2021) [51], Harirugsakul (2020) [28], Burr et al. (2012) [57], and Wu et al. (2005) [75] showed that smoking was significantly associated with reduced probability of utilizing dental services. However, Zhang et al. (2019) did not observe a significant relationship between smoking and visiting dentists by the elderly [20]. It could be stated that some elderly people visited dentists less often due to the shame of smoking and the damage they caused to their teeth. In fact, they were worried about the judgment of dentists regarding the use of nicotine. In addition, it seemed that people who smoked did not care about the health of their body organs, including their teeth, and as a result, their visits to dentists would decrease as well.

Physical problems were one of the components mentioned in the studies. According to the studies by Marino et al. (2014) and others, those who reported mobility problems visited dentists less often than the ones without such problems [17, 51, 79]. In other words, mobility problems as well as the obstacles to use stairs and wheelchairs, and the risk of falling in dental offices made it difficult for the elderly to utilize dental services [80].

Need for treatment was another sub-component of access. In this regard, Soares et al. (2021) and some other researchers showed that the elderly referred to dentists if they had pain or discomfort in their teeth [17, 24, 42]. It seemed that the elderly did not go to dentists for preventive care, but they did it only go when they needed medical care [81]. However, another study showed that the elderly did not go to dentists if they needed treatment [82]. This could be due to the low income of the elderly and the high costs of oral and dental services.

The most important sub-component of the mental factors main component was importance of care about dental health. The study by Manski (2016) et al. suggested that people who cared more about their oral health visited dentists more often [82]. It seems that the low priority given to oral and dental services [8] and the lack of understanding the necessity of oral and dental care in the elderly [35] could lead to decreased utilization of oral and dental services.

Unawareness about dental care was one of the important obstacles in utilization of the services, addressed in some of the studies [12, 19, 39, 77]. Appropriate knowledge about dental health could reduce disabling conditions such as chewing disorders in old age [83]. It seemed that the elderly, who had higher health literacy and knowledge, visited dentists more often for preventive checkups and oral care. Thus, providing education along with cooperation of various relevant bodies in terms of oral and dental health could help increase individuals’ awareness of oral and dental care [84, 85].

Fear was another important obstacle in utilization of oral and dental services. People who were afraid of the sound of dental handpieces [86] and had fear of dentists more often postponed or canceled their visits to dentists [45, 87]. However, a study showed that fear of dentistry decreased with age [88], the reason for which could be the fact that the elderly faced various diseases beyond teeth problems [89]. The difference in the results of the studies might be due to the individual characteristics of the elderly as well as their understanding and awareness of the importance of oral and dental care.

Traditional beliefs were one of the components mentioned in the studies. Mittal et al. (2019) showed that having traditional false beliefs such as “oral health is not part of physical health and toothless people do not need to see a dentist” led to decreased utilization of oral and dental services [12]. According to a study, Chinese elderly used more traditional self-care methods instead of professional methods to treat their oral health problems [90]. Another study reported that social discourses such as lack of interest in pursuing dental health, forgetfulness, unnecessary of paying for teeth and the need for saving income for children, and being at the end of life were among the things that reduced motivation to use dental services in the elderly [19]. Hence, it seems that knowing people’s traditional beliefs and trying to properly educate them about these beliefs can improve utilization of oral and dental services.

Previous negative experiences were among the sub-components investigated in the studies. Smith et al. (2020) reported in their study that traumatic oral and dental experiences in childhood affected the motivation and ability to access dental care and social isolation [19]. Pain, discomfort, fainting, lightheadedness, embarrassment or having a personal problem with the dentist are some of the negative experiences that patients face [91]. Patients who had such experiences visited dentists less often [92]. It seems that establishing friendly communications and giving a sense of trust by dentists could reduce these experiences and help patients utilize dental services more peacefully [93].

### Strength and limitations

In the current scoping review, an attempt was made to collect and review all the studies sporadically mentioned about the factors affecting oral and dental services` utilization among the elderly worldwide. This scoping review has extensively and comprehensively reviewed studies worldwide since 2000. These results can be useful for researchers in the field of oral and dental health, aging, and health policymakers of most countries to increase the use of oral and dental services by the elderly. In this study, the classification of demographic, social, psychological, access, and health level factors presented, being able to investigate various aspects affecting the use of oral and dental services in the elderly as much as possible. Also, the results of this study can be a start for other researchers’ local investigations in the field of factors affecting the elderly’s use of oral and dental services.

This study faced limitations in the databases and search strategies used by the researchers. In addition, due to the importance of the issue of oral and dental services utilization by the elderly, articles in this field are being updated. Therefore, additional studies are suggested to examine the appropriate measures and strategies taken by countries for each of the factors affecting utilization of oral and dental services by the elderly.

## Conclusion

The results showed that education level and income level were the most important factors affecting utilization of oral and dental services in the fields of demographic factors and access, respectively. Therefore, it is necessary to take necessary measures to increase the awareness of the society, especially the elderly, about the importance of oral and dental health, and to reduce the income gaps that affect utilization of these services. Considering social factors, the sub-component of living area had been discussed in most of the studies. People living in rural areas were facing more inequalities in utilization of oral and dental services due to the lack of facilities. Therefore, establishing dental clinics in villages and providing appropriate conditions for their sustainability could make it possible for villagers to use oral and dental services and to reduce injustice in utilization of such services. In the field of health level, the number of teeth was the factor that determined utilization of the services. Hence, the elderly should be informed that regular checkups could preserve the health of their remaining teeth. The importance of care and awareness of care were the most important mental factors. This indicates that the more the people pay attention to oral and dental health, the more checkups they will do. In addition, giving necessary information to the elderly in order to increase annual oral and dental checkups could help prevent tooth decay or loss.

### Electronic supplementary material

Below is the link to the electronic supplementary material.

Supplementary Material 1

## Data Availability

Data of this research is available and could be sent upon contact with the corresponding author.
