# Supplementary material for: Factors affecting oral and dental services` utilization among Elderly: a scoping review
Source: BMC Oral Health. 2023 Aug 27;23:597. doi: 10.1186/s12903-023-03285-4 (PMC10464329; doi:10.1186/s12903-023-03285-4)
Supplement: Supplementary file 1 — Supplementary Material 1 [file 12903_2023_3285_MOESM1_ESM.docx]

**Appendix**

See Tables A1 and A2.

**Table A1** A draft chart of data extraction

| **General Information** |
| --- |
| General I **Title of the manuscript** |
| Article No Language  Year of the publication First author  Place (Country) Corresponding  Type of article Journal name |
| **Article Characteristics** |
| Aims of the study  Study approach Study design |
| **Methodology** |
| Sampling Method Study Environment  Data collection Study Population  Data Analysis Sample Size |
| **Results** |
| Main results  Conclusion  Recommendations  Limitations |

**Table A2** Selected Studies on Factors Affecting Oral and Dental Services Utilization among Elderly

| N | Author | Title | Year | Place | Type | Participants | Results |
| --- | --- | --- | --- | --- | --- | --- | --- |
| 1 | Archuleta J [29] | The Impact of Education and Insurance Status on Past-Year Dental Visits Among Older Mexican Adults: Results From the 2001 and 2012 Mexican Health and Aging Study | 2022 | Mexico, North America | Original Article | Participants 50 years and older | - Increased dental visits in 50-year-old adults with high education and insurance |
| 2 | Hartmann C [62] | Factors associated with the use of dental services in the previous 12 and 36 months by Brazilian older people residing in rural areas | 2022 | Mexico, North America | Original Article | Participants older adult | - Lack of timely use of oral and dental health services among old people with non-white skin, who did not have health insurance |
| 3 | Wu YY [63] | Disparities in Dental Service Use among Adult Populations in the United States | 2022 | United States | Original Article | Participants among 3 age groups: 20-49, 50-64, and older adults more than 65-year-old | - Older black adults were less likely to use dental services |
| 4 | Drachev SN [42] | Prevalence of and factors associated with dental service utilization among early elderly in Lithuania | 2022 | Lithuania, Europe | Original Article | elderly aged 65-74 years | - Increased use of dental services in people with high education - Lower number of missing teeth were also significant predictors of dental service utilization. - Experiencing pain or discomfort in teeth or mouth was a significant predictor of having a dental visit in the last year. |
| 5 | Schwendicke F [102] | Dental service utilization in the very old: an insurance database  analysis from northeast Germany | 2021 | German | Original Article | Participants 75 years of age and older | - Decreased use of dental services (examination, prevention, surgery, and restorations) with age - More use of dental services in cities |
| 6 | Soares G.H [24] | Factors associated with utilisation of dental services by the elders from São Paulo, Brazil | 2021 | Brazil | Original Article | Participants 15-19, 35-44 and 65-74 years or more | - More likely to report utilization of dental services in older adults who rated their negative oral health-related quality of life, had their last dental appointment in the public health system, and sought treatment due to pain or lack of teeth - Having visited a dentist in the previous year was associated with education, income, age, ethnicity, and higher Family Health Strategy coverage in metropolitan areas |
| 7 | Patel N [99] | Prevalence of Past-Year Dental Visit Among  US Adults Aged 50 Years or Older, With  Selected Chronic Diseases, 2018 | 2021 | United State | Research Brief | Participants 50 years of age and older | - Lower dental service utilization among older adults with lower income, lower education, and no health care coverage and among those who smoked |
| 8 | Moeller J [51] | Dental care use and other population characteristics  of older Americans with self-reported chronic conditions  in the health and retirement study | 2021 | United State | Original Article | Participants 50 years of age and older | - Positive associations between regular utilization of dental care with having a college education, living in a high-income family, not smoking at all, not having certain functional limitations, and being under 65 years of age |
| 9 | Xu M [98] | Measuring and decomposing socioeconomic-related inequality  in the use of oral health services among Chinese adults | 2021 | China | Original Article | Participants 35-44, 55-64 and 65-74 years | - Most socioeconomic-related inequality in utilization of oral health services among adults aged 55-64 and 65-74 years with low income and no insurance |
| 10 | Xu M [69] | Factors associated with oral health service utilization among adults and older adults in China, 2015‐2016 | 2020 | China | Original Article | Participants 35-44 and 65-74 years | - More use of oral health services by women, insured people, those with high education level, those with high income level |
| 11 | Harirugsakul P [28] | Social backgrounds, oral behaviors  and dental service utilization  among Thai older adults: data from  the national oral health survey | 2020 | Thailand | Original Article | Participants 60-74 years | - Less use of dental services by rural elderly, low-income individuals, low-education ones, smokers, and those having Universal Coverage Scheme (UCS) |
| 12 | Choi J.S [18] | The Impact of Expanded National Health Insurance  Coverage of Dentures and Dental Implants on Dental  Care Utilization among Older Adults in South Korea: A Study Based on the Korean Health Panel Survey | 2020 | Korea | Original Article | Participants 65 years of age and older | - With increasing education, utilization of dental care and dental implants would increase. - With the decrease of household income, utilization of dental services and dental implants would decrease. |
| 13 | Smith BJ [104] | Longitudinal analysis of cost and dental  utilization patterns for older adults in  outpatient and long-term care settings in  Minnesota | 2020 | Minnesota, United States | Original Article | Participants 65 years of age and older | - Lowest use of dental services by patients without teeth |
| 14 | Smith L.A [19] | Barriers and enablers for dental care among dentate  home-based older New Zealanders who receive living support | 2020 | New Zealand | Original Article | Participants 65 years of age and older | - The most common obstacle to dental services was the cost of dental care. - Barriers to dental care: transportation, traumatic dental experiences in childhood, use of community-wide discourses on aging (no need, no interest, forgetfulness, not much life left, stop spending money and save it for children). Public health concerns affected the motivation and ability to access dental care and caused social isolation. - Facilitators of access to dental care: savings, family support, being a woman (paying more attention to dental health), awareness of the relationship between oral health and general health. |
| 15 | Limpuangthip N [77] | Predisposing and enabling factors associated with public denture service  utilization among older Thai people: a cross-sectional population-based study | 2019 | Thailand | Original Article | Participants60 years of age and older. | - Reduced utilization of dentures in older people working in agriculture compared to other working groups. - Reduced utilization of dentures in the elderly with high awareness and those visited by rural health volunteers - Increased use of dentures in the insured elderly - High use of denture services in the middle-aged and elderly with low education - Increased utilization of dentures in people with higher incomes - Women and highly educated people were more likely to use dentures**.** |
| 16 | Jang Y [101] | Factors Associated with Dental Service Use of Older Korean Americans | 2019 | Korean immigrants in California, New York, Texas, Hawaii, and Florida | Original Article | Participants 60 years of age and older | - Increased referrals to dentists with higher levels of education, problems with teeth or gums, coverage of dental health insurance, longer stays in the United States, and larger family networks |
| 17 | Mittal R [12] | Factors affecting dental service utilization among older Singaporeans eligible for subsidized dental care – a qualitative study | 2019 | Singapore | Original Article | Participants 65 years of age and older | - Traditional misconceptions about going to the dentist: oral health is not part of physical health; toothless people do not need to see a dentist; fear anxiety, previous negative experience, and lack of awareness |
| 18 | Spinler K [13] | Prevalence and determinants of dental  visits among older adults: findings of a nationally representative longitudinal study | 2019 | Germany | Original Article | Participants 45 years of age and older | - Decreased dental visits with age increased in the elderly |
| 19 | Zhang Wei [20] | Racial/Ethnic Disparities in Dental Service Utilization for Foreign-Born and U.S.-Born Middle-Aged and Older Adults | 2019 | United States | Original Article | Participants 50 years of age and older | - More use of dental services in the white race - Reduced utilization of dental services in people around 80 years of age and increased utilization of dental services in the US-born for all racial / ethnic groups - More use of dental services in women, insured people, married individuals, non-smokers, people with higher education and income, people with fewer disabilities in activities of daily living (ADL) and instrumental activities of daily living (IADL) |
| 20 | Thompson C [52] | Sinking Teeth into Dental Health of Older Adults in Jamaica | 2018 | Jamaica, India | Original Article | Participants 60 years of age and older. | - Increased tooth loss in people without health insurance - Increased dental visits with increasing level of education - Increased dental visits in women, middle aged (60-69 years), and urban people with health insurance |
| 21 | Olerud E [47] | Experience of dental care, knowledge and attitudes of older immigrants in Sweden-A qualitative study | 2018 | Sweden | Original Article | Participants 59-88 years | - Barriers to dental care for migrants were costs, language problems, and lack of trust in dental services. |
| 22 | Bommireddy VS [45] | Dental Service Utilization: Patterns and Barriers among Rural Elderly in Guntur District, Andhra Pradesh | 2016 | Andhra Pradesh, India | Original Article | Participants 55-86 years | - Fear was one of the most important barriers to dental services utilization |
| 23 | Manski R.J [82] | Differences Among Older Adults in the Types of Dental Services Used in the United States | 2016 | United States | Original Article | Participants 55 years of age and older | - No use of dental services in non-white people (the black, Hispanic, or other people) - No use of dental services in people with lower than high school education - lower use of oral services in people with dentures and people who had lost their teeth - More use of oral services in people with better oral health - Increased use of dental services in people with dental insurance, higher income and more wealth - lower use of regular care for prevention and diagnosis in people with lower oral health - No visit to dentists by people in need of dental care |
| 24 | Bíró A [100] | Supplementary private health insurance and health  care utilization of people aged 50+ | 2014 | Europe | Original Article | Participants 50 years of age and older | - Increased referrals to dentists with supplemental PHI coverage |
| 25 | Marino R.J [17] | Pattern and factors associated with utilization of dental  services among older adults in rural Victoria | 2014 | Victoria, Australia | Original Article | Participants 55 years of age and older | - Barriers to Dental Services: Cost of Services, Fear of Dentists, Length of waiting list, and Access to Oral Health Care Services - Better attitudes and more attention paid to oral health in people who visited a dentist - Decreased dental visits in toothless people and single individuals - More visits to dentists in women - Lower visits to dentists in people with mobility problems - Increased dental visits in people with greater understanding of oral health needs - Lower visits to dentists in people with less than high school education - Fewer visits to dentists in people whose source of income was business or assets than people whose source of income was investments and pensions |
| 26 | Ferreira Cde, O [23] | Factors associated with the use of dental services by elderly Brazilians | 2013 | Brazil | Original Article | Participants, 65-74 years | - Increased use of dental services with increased levels of education and income - Reduced use of dental services in the elderly with 0 to 20 teeth - Increased likelihood of seeing a dentist in the elderly in need of treatment or prosthesis |
| 27 | Somkotra T [94] | Inequality in oral health-care utilization exists among older Thais despite a universal coverage policy | 2013 | Thailand | Original Article | Participants 60 years of age and older. | - Increased use of oral health care in high-income elderly |
| 28 | Astrom AN [58] | Use of dental services  throughout middle and early old  ages: a prospective cohort study | 2013 | Sweden | Original Article | Participants 50-65 years | - Decreased regular use of dental health services in people aged 50- 65, increased regular use of dental services in groups with high socioeconomic status, among people with more remaining teeth, those who understood oral problems, and those who took more care of their teeth - Increased regular use of dental services in women, natives, and married people |
| 29 | Burr J.A [57] | Social Relationships  and Dental Care  Service Utilization  Among Older Adults | 2013 | United States | Original Article | Participants 51 years of age and older | - The association between not going to a dentist and being non-Hispanic black or Hispanic, having less SES, having more health concerns, and smoking more - With increased age, the likelihood of seeing a dentist increased - Women visited dentists more than men did. - More likely to see a dentist with higher education, higher household income, being married, and having dental insurance - lower visits to dentists in smokers and those with more restrictions in daily life activities - lower visits to dentists in people with the poorest health status who had fewer social relationships |
| 30 | Niesten D [35] | The impact of frailty on oral care behavior of  older people: a qualitative study | 2013 | Netherlands | Original Article | Participants 65 years of age and older | - Lack of belief in dental visits and tooth cleaning - Not caring about oral health and oral care - Older people often cut out oral care due to the distance, i.e., their dentists were too far. - The elderly often discontinued oral care because of disorientation and inconvenience of social support |
| 31 | MacEntee M.I [76] | Discussions on oral health care among elderly  Chinese immigrants in Melbourne and Vancouver | 2012 | Melbourne and Vancouver | Original Article | Participants 65 years of age and older | - Difficult access to dentistry due to immigrant language problems and financial costs - The important role of inconvenient transportation and cultural barriers to dental access |
| 32 | Allin S [95] | Measuring Socioeconomic Differences in Use of Health Care Services by Wealth Versus by Income | 2009 | 11 European nations: Austria, Belgium,  France, Denmark, Greece, Germany, Italy, the  Netherlands, Spain, Sweden, and Switzerland | Original Article | Participants 50 years of age and older. | - Increased probability of dental visits by increasing the level of education in most countries - Increased likelihood of seeing a dentist in wealthy people |
| 33 | Brothwell D.J [50] | Dental Service Utilization by Independently Dwelling Older Adults in Manitoba, Canada | 2008 | Manitoba, Canada | Original Article | Participants 65 years of age and older | - More dental visits in dentulous old people than in edentulous ones - More dental visits in dentulous people with higher education, main supporter, fewer restrictions in daily activities, more utilization of health services, and living in the city center - More dental visits in edentulous people with higher education, longer use of dentures, higher income, and more use of health services |
| 34 | Lai DW [73] | Use of Dental Care by Elderly Chinese Immigrants  in Canada | 2007 | Canada | Original Article | Participants 55 years of age and older | - Increased likelihood of using dental care by migrating from Hong Kong, living longer in Canada, having higher social support, and having a dental problem - Reduced use of dental care by increasing aging |
| 35 | Skaar D.D [97] | Demographic Factors Associated with Dental Utilization Among Community Dwelling Elderly in the United States, 1997 | 2006 | United States | Original Article | Participants 65 years of age and older | - More use of dental services by the white race - More visits to dentists in older people with higher incomes and those with a university degree - More visits to dentists in the elderly living in metropolitan areas - Lower visits to dentists in the older widows, divorced, and separated - More use of prevention services and less oral surgery services in the elderly with higher incomes - Lower use of dental visits with age increasing |
| 36 | Marino R [39] | Factors associated with self-reported use of dental health services among  older Greek and Italian immigrants | 2005 | Melbourne, Australia | Original Article | Participants 55 years of age and older | - Barriers to dental care in Greek: Length of waiting list, and in Italian: cost, Length of waiting list, and language barriers - Fewer visits to dentists in people with lower education - Reduced dental visits in people with moderate knowledge about oral health - The people who had the highest number of visits to dentists were those who had more than 20 teeth and stated that they did not work outside home or did not work in any jobs, other than workers before retirement. - Most likely to see a dentist were high-income Italians with higher knowledge of oral health and those under 70 years of age. |
| 37 | Wu B [75] | Comparison of Utilization of Dental Care Services Among  Chinese- and Russian-Speaking Immigrant Elders | 2005 | Boston, United States | Original Article | Participants 60 years of age and older | - More visits to dentists in older people with a university degree, longer stay in the US, higher social interactions, and fewer visits to dentists in older smokers - Decreased dental visits with age increasing and in people with dentures - Income was found to be negatively associated with utilization of dental services. |
| 38 | Adams C [53] | Dental visits in older Western Australians: A comparison of  urban, rural and remote residents | 2004 | Australia | Original Article | Participants, 60 years of age and older. | - Most visits to dentistry in cities and the least in remote areas. - Decreased dental visits with age increasing. - Difficult access to facilities and appointments for villagers and, especially, for older people in remote areas |
| 39 | Adut R [103] | Past and Present Geographic Location as Oral Health  Markers Among Older Adults | 2004 | Occupied Palestine | Original Article | Participants, 65 years of age and older. | - Tendency to use dental services in urban older people |
| 40 | Manski R.J [96] | Dental Insurance Visits and Expenditures  Among Older Adults | 2004 | United States | Original Article | Participants 55 years of age and older | - Elderly with teeth had more insurance coverage - Less dental coverage in poor, low-income, and middle-income people - Increased referrals to dentists in the age group 65-74 years; but in the oldest group (75 years and above) the probability of referral was lower - Reduction of dental visits in poor and low-income elderly people compared to richer elderly people - Increased dental visits in women, university graduates, and high school graduates |
| 41 | Ahluwalia K.P [8] | Oral disease burden and dental services utilization by Latino and African-American seniors in Northern Manhattan | 2003 | Harlem and Washington Heights, New York City | Original Article | Participants 55-95 years old | - The most important reasons for not going to the dentist were lack of teeth and low priority of dental treatment - Increased use of dental services in people with over eight years of education and dentulous people |
| 42 | Avlund K [79] | Functional Ability and Oral Health Among Older People: A Longitudinal Study from Age 75 to 80 | 2001 | Copenhagen County, Denmark | Original Article | Participants 50-80 years | - People of 75 years of age and older had dysfunction of teeth, more chewing problems, and less use of dental services - Elderly people with low mobility were 1.7 times more likely to have no teeth / fewer teeth and 1.5 times more likely to not use dental services regularly. - Lack of teeth, difficulty in chewing, and regular use of dental services with reduced education and income - Increased risk of edentulousness and non-use of dental services in men |
